# Short residence duration was associated with asthma but not cognitive function in the elderly: USA NHANES, 2001–2002

**DOI:** 10.1007/s11356-016-7850-3

**Published:** 2016-10-18

**Authors:** Ivy Shiue

**Affiliations:** 1 Faculty of Health and Life Sciences, Department of Healthcare, Northumbria University, Newcastle upon Tyne, England NE1 8ST UK; 2 Owens Institute for Behavioral Research, University of Georgia, Athens, USA; 3 Alzheimer Scotland Dementia Research Centre, University of Edinburgh, Edinburgh, UK

**Keywords:** Housing, Cognitive function, Risk factor, Aging, Chronic disease, Asthma

## Abstract

There has been a growing interest in how the built environment affects health and well-being. Housing characteristics are associated with human health while environmental chemicals could have mediated the effects. However, it is unclear if and how residence duration might have a role in health and well-being. Therefore, the aim of the present study was to investigate the associations among residence duration, common chronic diseases, and cognitive function in older adults in a national and population-based setting. Data were extracted from the US National Health and Nutrition Examination Survey, 2001–2002, with assessment information on demographics, lifestyle factors, housing characteristics, self-reported common chronic diseases, and cognitive function by using the digit symbol substitution test from the Wechsler Adult Intelligence Scale (a measurement of attention and psychomotor speed). Statistical analyses including the chi-square test, *t* test, and survey-weighted general linear modeling and logistic regression modeling were performed. Residence duration was significantly associated with risk of asthma but not with other chronic disease, showing a longer stay in the same housing leading to lower risk of asthma (OR 0.43, 95%CI 0.27–0.69, *P* = 0.002) among the American older adults. However, having asthma was not associated with cognitive function decline. In conclusion, residence duration was found to be associated with risk of asthma but not cognitive function. Future research examining the relationship of residence duration and cognitive tests by other domains of cognitive function following asthma episodes would be suggested. For practice and policy implications, familiarity with the housing environment might help with lessening respiratory symptoms.

## Introduction

### Evidence before this study

There has been a growing interest in how the built environment, including our own homes, affects our health and well-being in recent years (Mitty 2010). Early animal studies observed that the defeated and subsequently individually housed rats displayed impaired social memory, decreased social interaction, and diminished anticipation for a sucrose solution for up to a period of 3 months (Von Frijtag et al. 2000), and cognitive deficits were followed by an interaction of genotype and housing environment in rodent models of schizophrenia (Turner and Burne 2013) or Alzheimer’s disease (Pietropaolo et al. 2014; Ambrée et al. 2006) to affect learning and memory functioning (Marques et al. 2009; Sonninen et al. 2006). In human studies, housing instability in the 12 months prior to baseline was found to predict lower verbal cognitive abilities across all age groups (Fowler et al. 2015), likely through the mechanisms of reward (”optimism”) or punishment (“pessimism”) systems (Parker et al. 2014), parental disruption (Coley et al. 2013), or relocation controllability and adjustment (Bekhet and Zauszniewski 2013). Some of the relocated nursing home residents also demonstrated significant higher levels of salivary cortisol and lower depression, anxiety, and pulse rates than those who had not yet moved (Hodgson et al. 2004). However, this might depend on extensive relocation preparation and support to diminish the stress of relocation over time and across diagnostic categories (Lander et al. 1997; Engle 1985).

### Knowledge gap

Housing inequalities could pose significant social and health problems in many societies. Several studies have investigated the effects of housing characteristics on adult human health and biomarkers (Jacobs et al. 2009; Shiue and Shiue 2003), with more literature focusing on children. Even environmental chemicals could have a role in mediating the housing effect on adult health conditions (Shiue and Bramley 2015). However, there has been limited consideration of the potential effect of residence duration, a probabilistic risk assessment method in establishing the distribution of exposure in a population (Sedman et al. 1998), on human health including chronic diseases and cognitive function.

### Study aim

**Fig. 1:**
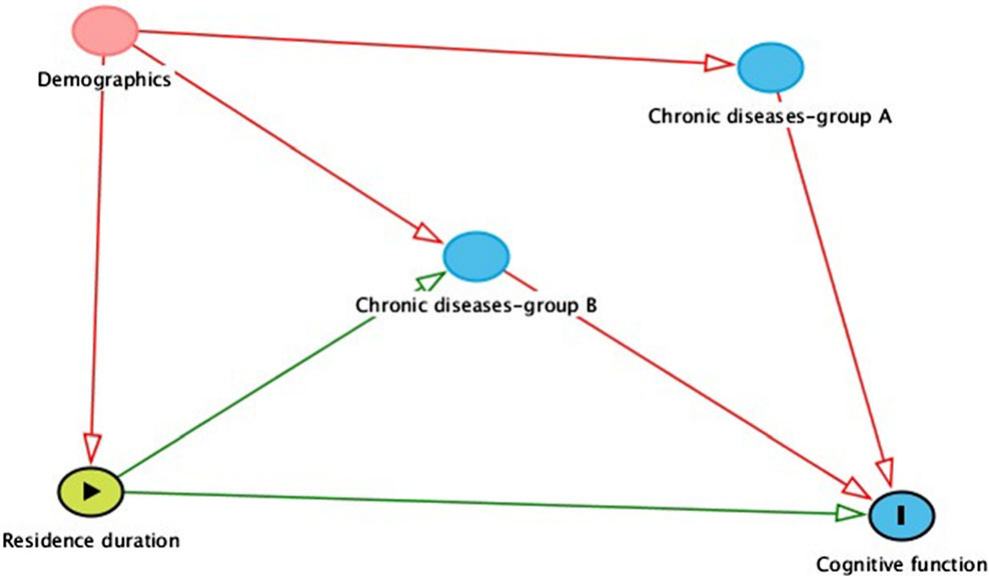
Pathway of residence duration, chronic disease, and cognition

Following this context, therefore, the aim of the present study was to investigate the associations among residence duration, common chronic diseases and cognitive function (see the illustrated pathway in Fig. 1) in older adults in a national and population-based setting.

## Method

### Study sample and variables

The US National Health and Nutrition Examination Survey (NHANES) has been a national, population-based, multi-year, cross-sectional study representative of the entire American population. For the current analysis, data from the 2001 and 2002 (more details on the sampling method and procedure via http://wwwn.cdc.gov/nchs/nhanes/search/datapage.aspx?Component=Questionnaire&CycleBeginYear=2001) that were with cognitive function assessment were retrieved. In other words, there was no cognitive function measured after 2002, so no recent data could be used for analysis. Moreover, only older adults aged 60 and above were included in the present study because the cognitive function was measured in this age group only.

Information on demographics (more details via http://wwwn.cdc.gov/nchs/nhanes/search/datapage.aspx?Component=Demographics&CycleBeginYear=2001), residence duration (question: How many years have you lived at this address? more details via http://wwwn.cdc.gov/nchs/nhanes/2001-2002/HOQ_B.htm), self-reported chronic diseases (question: Has a doctor or other health professionals ever told you that you have X disease? more details via http://wwwn.cdc.gov/nchs/nhanes/2001-2002/MCQ_B.htm) and cognitive function (the digit symbol substitution test from the Wechsler Adult Intelligence Scale, a measure of attention and psychomotor speed, DSS) was obtained by a household interview (The Psychological Corporation 1997; more details via http://wwwn.cdc.gov/nchs/nhanes/2001-2002/CFQ_B.htm), which has been widely used and subsequently published (Shiue and Starr 2012).

### Statistical analysis

**Fig. 2:**
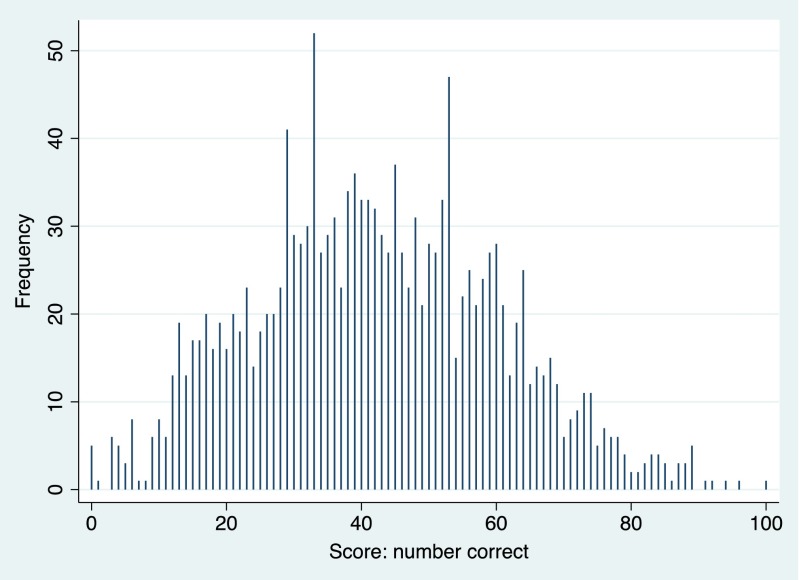
Distribution of digit symbol score with correct answers in the elderly aged 60+

**Fig. 3:**
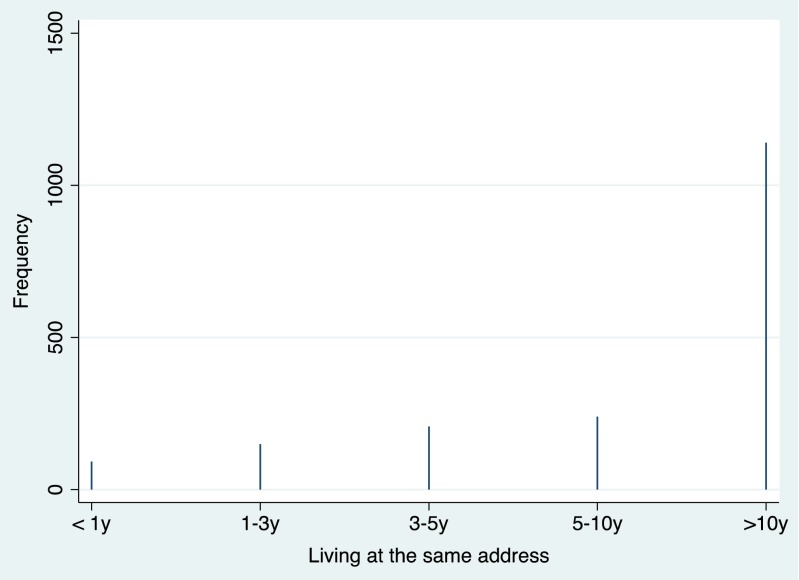
Distribution of housing residing duration in the elderly aged 60+

In the first step of analysis, distribution of digit symbol scores and of residence duration was presented by using spike plots (see Figs. 2 and 3). In the second step of analysis, associations between common chronic diseases and cognitive function were examined by using the *t* test. In the third step, associations between housing duration, common chronic disease, and digit symbol scores were examined by using the chi-square test and survey-weighted generalized linear models or logistic regression models depending on the study outcome being continuous or categorical. Effects were shown in odds ratios with 95 % confidence intervals, with *P* < 0.05 as statistically significant. Covariates including age, sex, education level, vitamin D level, cholesterol level, smoking habit, and physical activity level were adjusted in the statistical models. STATA statistical software version 13.0 (STATA, College Station, Texas, USA; more details via http://www.stata.com/) was used to perform all the statistical analyses.

### Ethics considerations

Since there were only secondary data analyses employed without any participant’s personal information identified by extracting statistical data from the UK Data Archive website in the present study, no further ethics approval for conducting the present study was required (more details via http://www.ethicsguidebook.ac.uk/Secondary-analysis-106).

## Results

Associations between common chronic diseases and cognitive function are shown in Table 1. Apparently, people with previous stroke, heart attack, heart failure, diabetes, or trouble seeing had lower digit symbol scores. Residence duration was significantly associated with risk of asthma but not with other chronic diseases (see Table 2), showing a longer stay in the same housing leading to lower risk of asthma (OR 0.43, 95%CI 0.27–0.69, *P* = 0.002) among the American older adults. However, having asthma was not associated with cognitive function decline.

**Table 1:** Associations of chronic diseases and digit symbol score (*n* = 1558)

Prior health events		Present	Absent	*P* value
	*n*	Mean (SD)	Mean (SD)	
Asthma	124/1557	41.7 (18.8)	42.2 (18.2)	0.769
Arthritis	723/1556	41.6 (18.1)	42.7 (18.3)	0.230
Stroke	105/1552	31.1 (15.8)	43.0 (18.1)	*<0.001*
Heart attack	165/1550	35.0 (14.3)	42.8 (18.3)	*<0.001*
Emphysema	61/1554	38.5 (15.5)	42.3 (18.3)	0.107
Heart failure	105/1543	38.1 (17.3)	42.7 (18.3)	*0.002*
Coronary heart disease	158/1535	42.8 (16.6)	42.3 (18.4)	0.746
Angina	118/1538	39.8 (15.2)	42.5 (18.4)	0.118
Chronic bronchitis	97/1555	41.0 (15.6)	42.3 (18.4)	0.489
Liver problem	49/1553	41.2 (18.5)	42.2 (18.2)	0.705
Cancer	322/1556	43.8 (17.4)	41.8 (18.4)	0.089
Diabetes	265/1517	37.1 (16.8)	43.2 (18.4)	*<0.001*
Blood transfusion	351/1511	41.8 (16.4)	42.2 (18.8)	0.702
Trouble seeing	358/1556	36.3 (17.4)	43.9 (18.1)	*<0.001*

**Table 2:** Associations of residence duration, chronic diseases, and digit symbol score (*n* = 1558)

	≤10years (*n* = 688, 37.6 %)	>10y (*n* = 1140, 62.4 %)	*P* value	OR (95%CI)*	*P* value
Digit symbol score	41.2 (18.4)	43.0 (18.1)	0.067	–	–
Asthma	76/687	77/1140	*0.001*	*0.43 (0.27–0.69)*	*0.002*
Arthritis	302/688	552/1137	*0.054*	1.13 (0.79–1.61)	0.478
Stroke	62/685	97/1136	0.708	1.00 (0.56–1.79)	0.994
Heart attack	78/684	124/1134	0.758	0.91 (0.53–1.56)	0.720
Emphysema	37/683	37/1138	*0.023*	0.50 (0.23–1.07)	0.072
Heart failure	62/683	79/1126	0.113	0.97 (0.57–1.66)	0.898
Coronary heart disease	70/677	113/1117	0.880	0.94 (0.63–1.42)	0.765
Angina	57/677	85/1127	0.503	1.03 (0.52–2.05)	0.921
Chronic bronchitis	47/685	74/1138	0.766	0.99 (0.51–1.90)	0.969
Liver problem	24/684	30/1137	0.289	0.79 (0.28–2.20)	0.634
Cancer	127/685	255/1140	*0.052*	1.36 (0.99–1.88)	0.057
Diabetes	129/674	194/1108	0.386	0.91 (0.59–1.38)	0.624
Blood transfusion	174/670	284/1103	0.917	0.99 (0.75–1.31)	0.944
Trouble seeing	205/688	276/1138	*0.009*	0.73 (0.50–1.06)	0.091

*Adjusted for age, sex, education level, vitamin D level, cholesterol level, smoking habit, physical activity level, and survey weighting

## Discussion

### Housing, chronic diseases, and cognition

The linkage of vascular risk factors (including stroke, heart attack, and diabetes) and late-life cognitive decline has been well established (Tuligenga 2015; Carmichael 2014; Knopman et al. 2009) while that of heart failure and cognitive function has been unconfirmed (Cannon et al. 2015). Plausible underlying mechanisms might be related to cerebral hypoperfusion or occult cerebrovascular disease, and it seems likely that these may coexist and exert synergistic effects. Moreover, there is no specific treatment guidance in this (Cannon et al. 2015; Carmichael 2014). Recently, it was also observed that people with vision loss due to three different age-related eye diseases could have lower cognitive scores (Harrabi et al. 2015). The findings from the present study are consistent with those in the abovementioned literature.

The risk of asthma was related to the length of stay in older adults as observed in the present study, although from previous research, the effect seemed to be the opposite in children (Cabieses et al. 2014). Similarly, in previous animal studies, it was also observed that there was an inverse association between residence duration and cognitive impairment in polar environments (John Paul et al. 2010; Reed et al. 2001) or the dependent context (Missotten et al. 2009; Jackson 1974). One of the reasons in such contrast between the literature and the present study might be that older adults could have been better accustomed to the living environment for a longer period of time living in the same or similar environment.

### Strengths and limitations

The present study has a few strengths. Firstly, this exploratory study is the first to examine the associations among residence duration, common chronic disease, and cognitive function in the elderly aged 60 and above from the general population in a national setting. Secondly, many different types of common chronic diseases were able to be included. However, there are also limitations that cannot be ignored. First, cognitive function was only assessed by the digit symbol test while there are other tests to measure different domains of cognition along the life course. Second, only associations but not the causality can be established in the present study due to the cross-sectional observational study design in nature. Taken together, future research with a longitudinal approach plus other cognitive tests measuring other domains of cognitive function to confirm or refute the current observation would be warranted.

### Directions for future research, practice, and policy

In conclusion, residence duration was found to be associated with risk of asthma but not cognitive function. Future research examining the relationship of residence duration and cognitive tests by other domains of cognitive function following asthma episodes would be suggested. For practice and policy implications, familiarity with the housing environment might help with lessening respiratory symptoms.
